# Molecularly Guided Therapy and the Tumor Microenvironment in Advanced Cholangiocarcinoma: Current Evidence and Emerging Strategies Targeting FGFR2, HER2, and IDH1 with Pemigatinib, Zanidatamab, and Ivosidenib

**DOI:** 10.3390/jcm15197396

**Published:** 2026-09-23

**Authors:** Piotr Kawczak, Tomasz Bączek

**Affiliations:** 1 Department of Pharmaceutical Chemistry, Faculty of Pharmacy, Medical University of Gdańsk, 80-416 Gdansk, Poland; tomasz.baczek@gumed.edu.pl; 2 Department of Nursing and Medical Rescue, Institute of Health Sciences, Pomeranian University in Słupsk, 76-200 Slupsk, Poland

**Keywords:** CCA, precision medicine, targeted therapy, FGFR2, HER2, IDH1, pemigatinib, zanidatamab, ivosidenib, biliary tract cancer

## Abstract

Cholangiocarcinoma (CCA) remains an aggressive malignancy characterized by late-stage diagnosis, substantial molecular heterogeneity, and limited therapeutic options. Advances in molecular characterization have identified actionable alterations and expanded systemic treatment beyond conventional cytotoxic chemotherapy. This review examines three clinically relevant and mechanistically distinct targeted strategies in advanced CCA. Pemigatinib, a selective fibroblast growth factor receptor 1–3 (FGFR1–3) inhibitor, targets oncogenic signaling driven by FGFR2 fusions or rearrangements, which occur predominantly in intrahepatic CCA. Zanidatamab, a bispecific human epidermal growth factor receptor 2 (HER2)-directed antibody targeting two extracellular HER2 domains, provides a therapeutic approach for HER2-positive biliary tract cancers (BTCs). Ivosidenib, a selective inhibitor of mutant isocitrate dehydrogenase 1 (IDH1m), suppresses production of the oncometabolite D-2-hydroxyglutarate and targets the associated metabolic and epigenetic dysregulation in IDH1-mutant CCA. The review integrates the molecular rationale and pivotal clinical evidence supporting these therapies with their safety profiles, mechanisms of acquired resistance, biomarker selection, and therapeutic positioning. Particular attention is given to tumor heterogeneity and the tumor microenvironment as determinants of treatment response and resistance, as well as to emerging strategies involving next-generation inhibitors, antibody–drug conjugates, immunotherapy, rational combinations, and longitudinal molecular monitoring. Collectively, the clinical development of pemigatinib, zanidatamab, and ivosidenib demonstrates the therapeutic value of matching actionable molecular alterations with mechanism-based treatments, while highlighting the need to overcome resistance and optimize treatment sequencing to achieve more durable benefit in advanced CCA.

## 1. Introduction

CCA represents a biologically heterogeneous group of aggressive malignancies arising from the epithelial lining of the biliary tract and remains associated with poor prognosis and high mortality worldwide. Anatomically, CCA is classified into intrahepatic, perihilar, and distal subtypes, which differ in their molecular profiles, clinicopathological features, and therapeutic vulnerabilities [1,2,3,4]. Considerable intertumoral and intratumoral heterogeneity, driven by genetic, epigenetic, inflammatory, and microenvironmental factors, further contributes to variable clinical behavior and treatment response [5,6,7]. Despite advances in diagnosis and systemic therapy, most patients present with unresectable or metastatic disease and have poor long-term outcomes [8,9,10,11]. The increasing incidence of intrahepatic CCA reported in several geographic regions further underscores its clinical and public health significance [6,7].

Historically, systemic treatment of advanced CCA relied predominantly on cytotoxic chemotherapy, with gemcitabine plus cisplatin established as first-line standard of care following the pivotal ABC-02 trial (Advanced Biliary Cancer-02 trial) [12]. Although this combination improved overall survival (OS) compared with gemcitabine monotherapy, its benefit remained limited [12,13]. More recently, immune checkpoint inhibitors, which restore antitumor T-cell activity by blocking inhibitory immune signaling, have been incorporated into platinum-based frontline regimens. Nevertheless, disease progression remains common, sustaining the need for more effective systemic strategies [14,15,16]. These limitations have accelerated the development of molecularly targeted therapies, immunotherapeutic combinations, and biomarker-guided approaches [17,18,19].

Advances in next-generation sequencing (NGS), transcriptomic profiling, and molecular diagnostics have revealed a complex genomic landscape containing clinically actionable alterations [20,21,22,23]. These findings have supported a shift from exclusively empiric treatment toward biomarker-guided therapy [24,25,26,27]. The distribution of actionable alterations differs by anatomical subtype; intrahepatic CCA is particularly enriched for FGFR2 rearrangements and IDH1 mutations, whereas HER2 alterations are more frequently observed in extrahepatic disease [28,29,30,31]. Accordingly, comprehensive molecular profiling has become increasingly important for identifying therapeutic options and facilitating clinical trial enrollment [32,33].

FGFR2 fusions and rearrangements define one of the most clinically relevant molecular subsets of intrahepatic CCA. Aberrant FGFR signaling promotes proliferation, survival, angiogenesis, and resistance to apoptosis through downstream MAPK, PI3K/AKT, and JAK/STAT pathways [34,35]. Pemigatinib, a selective FGFR1–3 tyrosine kinase inhibitor, demonstrated clinically meaningful activity in previously treated CCA harboring FGFR2 fusions or rearrangements and became one of the first molecularly targeted therapies approved for this biomarker-defined population [36,37,38]. Earlier translational studies identifying FGFR signaling as a therapeutic vulnerability provided the biological rationale for subsequent FGFR-directed drug development [32,33,39].

HER2 amplification and overexpression represent another therapeutically relevant alteration, occurring more frequently in extrahepatic CCA and gallbladder cancer but also in selected intrahepatic tumors [40,41]. HER2 activation promotes tumor growth and survival through signaling pathways including PI3K/AKT and RAS/MAPK [42]. Following the established role of HER2-directed therapy in breast and gastric cancers, increasing evidence supports its therapeutic potential in BTCs [43,44]. Zanidatamab is a bispecific HER2-directed antibody that binds two distinct extracellular HER2 epitopes, promoting receptor clustering and internalization as well as immune-mediated antitumor activity [45,46]. Clinical studies have demonstrated meaningful and durable responses in HER2-positive BTCs, supporting HER2 as an actionable therapeutic target in this molecularly selected population [46,47].

IDH1 mutations, found predominantly in intrahepatic CCA, represent a distinct metabolic vulnerability [48]. IDH1m aberrantly converts α-ketoglutarate to the oncometabolite D-2-hydroxyglutarate, resulting in epigenetic dysregulation and impaired cellular differentiation [49]. Ivosidenib, a selective inhibitor of IDH1m, suppresses D-2-hydroxyglutarate production and has demonstrated improved progression-free survival (PFS) and clinically meaningful disease stabilization in previously treated IDH1-mutated CCA [50,51,52]. These findings established IDH1m as another therapeutically actionable driver in advanced CCA.

Despite these advances, several challenges limit the durability and broader effectiveness of biomarker-directed therapy, including acquired resistance, intratumoral heterogeneity, clonal evolution, access to molecular testing, and uncertainty regarding optimal treatment sequencing [24,26,53,54,55,56,57]. Resistance may arise through secondary FGFR mutations, activation of bypass signaling pathways, epigenetic adaptation, and tumor microenvironment-mediated escape [58,59,60,61]. The immunosuppressive CCA microenvironment, characterized by dense desmoplastic stroma, tumor-associated macrophages, cancer-associated fibroblasts, and immunoregulatory cytokine networks, further contributes to therapeutic resistance and represents an important area for therapeutic development [6,62].

Rational combination strategies integrating targeted agents with chemotherapy, immunotherapy, antibody–drug conjugates, and other immune-modulating approaches are therefore under active investigation [63,64,65,66,67]. Emerging technologies, including circulating tumor DNA (ctDNA) analysis and liquid biopsy, may improve detection of molecular evolution and facilitate treatment monitoring, while artificial intelligence-assisted biomarker discovery, imaging analysis, radiomics, and response prediction may further refine patient selection [68,69,70,71,72]. Together, these developments support an increasingly multidisciplinary approach to advanced CCA that integrates molecular and clinical information to guide therapeutic decisions [19,73,74].

This narrative review focuses on three mechanistically distinct therapeutic strategies that exemplify biomarker-directed treatment in advanced CCA: FGFR2 inhibition with pemigatinib, HER2-directed therapy with zanidatamab, and IDH1m inhibition with ivosidenib. The review integrates their molecular rationale, mechanisms of action, clinical evidence, resistance mechanisms, and emerging therapeutic strategies, with particular attention paid to interactions between molecular drivers, the tumor microenvironment, and treatment response.

A structured literature search of PubMed and Scopus was conducted for relevant English-language publications published between 2006 and August 2026. This period was selected to encompass key translational, preclinical, and clinical developments underlying molecularly targeted therapy in advanced CCA. Search terms included combinations of “cholangiocarcinoma,” “biliary tract cancer,” “pemigatinib,” “zanidatamab,” “ivosidenib,” “FGFR2,” “HER2,” “IDH1,” “targeted therapy,” “precision medicine,” “molecular profiling,” “combination therapy,” and “biomarker-driven therapy.”

Eligible evidence included phase II and III prospective clinical trials, randomized controlled trials, basket studies, registration-directed investigations, meta-analyses, translational studies, and clinically informative prospective cohort studies evaluating targeted or biomarker-guided therapies in CCA and other solid tumors harboring relevant actionable alterations. Priority was given to pivotal and regulatory-supporting studies of FGFR2-, HER2-, and IDH1-directed therapies. Selected retrospective and real-world studies were included when they provided clinically relevant information on efficacy, resistance, safety, molecular selection, or treatment sequencing.

Figure 1 illustrates a schematic overview of molecularly guided therapeutic strategies in advanced CCA, integrating molecular profiling, biomarker-driven patient stratification, and selected targeted therapeutic approaches involving FGFR2, HER2, and IDH1 inhibition with pemigatinib, zanidatamab, and ivosidenib, respectively, according to actionable genomic alterations, disease setting, and therapeutic sequencing principles.

## 2. Pemigatinib

Pemigatinib is an orally bioavailable, selective inhibitor of FGFR1, FGFR2, and FGFR3 that suppresses aberrant FGFR-mediated signaling involved in tumor proliferation, angiogenesis, survival, and resistance to apoptosis [75,76,77]. FGFR2 fusions and rearrangements represent among the most clinically actionable alterations in biliary tract malignancies, occurring predominantly in intrahepatic CCA in approximately 10–20% of cases and defining a distinct molecular and clinicopathological subgroup [28,29,78]. These rearrangements promote constitutive receptor activation and downstream MAPK, PI3K/AKT, JAK/STAT, and PLCγ signaling, creating an oncogenic dependency amenable to FGFR inhibition [34,55,79]. The development of pemigatinib consequently represented an important advance in biomarker-directed treatment of advanced CCA [3,80,81,82,83]. Figure 2 illustrates its mechanism of action.

Translational and preclinical studies demonstrated that FGFR inhibition suppresses proliferation and induces apoptosis in FGFR2-driven CCA models, providing the rationale for clinical development [27,31,85]. Initial clinical evidence emerged from the first-in-human phase I/II FIGHT-101 study (NCT02393248), which evaluated pemigatinib in advanced solid tumors with FGFR alterations or pathway dysregulation [86]. The study established the recommended phase II dose and demonstrated preliminary antitumor activity, with responses enriched in tumors harboring FGFR2 fusions or rearrangements [36,86]. Despite the heterogeneity and small cohorts inherent to an early-phase basket study, FIGHT-101 provided clinical support for FGFR2 rearrangement as a predictive biomarker in CCA [36,86,87]. Structural and pharmacological studies subsequently characterized pemigatinib selectivity and identified kinase-domain mutations as potential mechanisms of resistance [88].

The pivotal multicenter, open-label phase II FIGHT-202 trial (NCT02924376) evaluated pemigatinib in previously treated advanced, metastatic, or unresectable CCA [24]. In patients with FGFR2 fusions or rearrangements, the final analysis demonstrated an objective response rate (ORR) of 37.0%, median duration of response (mDOR) of 9.1 months, median progression-free survival (mPFS) of 7.0 months, and median overall survival (mOS) of 17.5 months [89]. Substantially less activity was observed in tumors harboring other FGF/FGFR alterations [24,26,90]. These findings supported regulatory approval and established pemigatinib as a treatment option for previously treated FGFR2 fusion/rearrangement-positive CCA [24,83,89,91].

FIGHT-202 nevertheless had important limitations, particularly its single-arm design and absence of a randomized comparator [24,89]. The trial also enrolled a selected population with preserved performance status and access to molecular testing, potentially limiting generalizability to routine practice [92]. Despite these limitations, the magnitude and durability of response were clinically relevant in a previously treated population with limited therapeutic options [63] and further illustrated the molecular heterogeneity of CCA [20].

The randomized phase III FIGHT-302 trial (NCT03656536) subsequently compared first-line pemigatinib with gemcitabine plus cisplatin in patients with advanced CCA harboring FGFR2 rearrangements [12,93]. Pemigatinib significantly prolonged median PFS compared with chemotherapy (8.3 vs. 6.8 months; HR, 0.58; 95% CI, 0.39–0.87), increased the ORR (47% vs. 15%), and prolonged median DOR (14.2 vs. 6.3 months), whereas median OS was similar between groups (24.4 vs. 25.0 months) [94]. Interpretation of the OS findings should consider subsequent FGFR inhibitor treatment in the chemotherapy group and the early closure of FIGHT-302 following changes in the first-line standard of care [94]. Nevertheless, the trial provides randomized evidence of substantial antitumor activity with FGFR2-directed therapy in the first-line setting. Questions regarding optimal sequencing and integration with contemporary chemoimmunotherapy remain unresolved [95,96]. Early studies combining pemigatinib with gemcitabine/cisplatin have begun to investigate such approaches, although evidence remains preliminary [97].

Acquired resistance to FGFR inhibition is biologically heterogeneous and may involve secondary FGFR2 kinase-domain mutations, including gatekeeper alterations, activation of bypass signaling pathways, epithelial–mesenchymal transition, and adaptive pathway reprogramming [98,99,100]. Polyclonal FGFR2 resistance mutations can be detected using ctDNA, illustrating the potential utility of liquid biopsy for longitudinal assessment of clonal evolution [99,100,101]. These observations have stimulated interest in sequential FGFR inhibition and next-generation agents such as futibatinib, an irreversible FGFR1–4 inhibitor capable of retaining activity against selected resistance mutations [102]. Additional preclinical mechanisms implicated in pemigatinib resistance include ferroptosis-related remodeling through the ROCK2/UBA52/DRP1 axis, m6A-mediated ARRB2 upregulation affecting MAPK and Hippo signaling, and autophagy-related VPS33A signaling [103,104,105]. Collectively, available evidence indicates that both on-target and off-target mechanisms contribute to resistance in FGFR2-rearranged BTC [106].

Beyond tumor-intrinsic genomic mechanisms, the TME may further shape resistance to FGFR inhibition. FGFR2-altered CCA has been associated with a relatively non-inflamed immune phenotype, including reduced antigen-presentation programs and immune-cell infiltration, suggesting that FGFR2-driven disease may coexist with immune exclusion [107]. In parallel, the desmoplastic CCA microenvironment, comprising cancer-associated fibroblasts, extracellular-matrix components, and immunosuppressive cell populations, can provide paracrine and adaptive survival signals that may interact with tumor-cell-intrinsic resistance pathways [108,109]. Resistance to selective FGFR inhibitors is characterized not only by polyclonal secondary FGFR2 kinase-domain mutations but also by activation of alternative signaling pathways, including PI3K/mTOR and MAPK signaling [110]. Such adaptive signaling may interact with stromal and immune-mediated survival cues, potentially supporting treatment-resistant residual disease [109]. Although the direct contribution of individual TME components to clinical pemigatinib resistance remains incompletely defined, these findings support a model in which clonal evolution and microenvironmental adaptation may jointly sustain resistance to FGFR-directed therapy.

In this context, an emerging strategy for overcoming the limitations of earlier FGFR inhibitors is the development of agents with greater FGFR2 isoform selectivity. Lirafugratinib (RLY-4008) is a highly selective, irreversible FGFR2 inhibitor designed to maintain potent FGFR2 suppression while limiting inhibition of other FGFR family members. Preclinical studies demonstrated >250-fold and >5000-fold selectivity over FGFR1 and FGFR4, respectively, together with activity against several FGFR2 kinase-domain mutations associated with resistance to earlier FGFR inhibitors [111,112]. This selectivity is potentially clinically relevant because FGFR1 inhibition contributes to the characteristic hyperphosphatemia associated with less selective FGFR inhibitors, whereas selective FGFR2 targeting may permit sustained target inhibition while reducing off-isoform toxicity [111,112]. Furthermore, lirafugratinib retains activity against multiple FGFR2 mutations that confer resistance to pan-FGFR inhibitors, providing a rationale for molecularly guided sequential FGFR inhibition [111,113]. Nevertheless, FGFR2-selective inhibition does not eliminate acquired resistance. Recent clinical analyses identified polyclonal FGFR2 kinase-domain mutations and receptor tyrosine kinase–mitogen-activated protein kinase (RTK–MAPK) bypass alterations as important mechanisms of resistance to lirafugratinib [113]. Thus, next-generation FGFR2-selective inhibitors represent a promising evolution of FGFR-targeted therapy rather than an established replacement for pemigatinib, and their comparative clinical benefit requires confirmation in prospective studies.

Combination strategies are being investigated to delay or overcome resistance, including FGFR inhibition with immune checkpoint blockade, antiangiogenic therapy, MEK inhibition, or chemotherapy [63,95,114]. Immune checkpoint inhibitors target inhibitory pathways that constrain antitumor T-cell activity, whereas MEK inhibitors suppress downstream MAPK signaling. In view of the immune-excluded phenotype described above, combinations incorporating immune checkpoint blockade provide a mechanistically plausible strategy, although clinical evidence remains limited and questions concerning toxicity, sequencing, and patient selection remain unresolved [114,115,116,117,118]. A reported case of pemigatinib combined with sintilimab, an anti-PD-1 immune checkpoint inhibitor, followed by resection in recurrent FGFR2-positive intrahepatic CCA illustrates the potential of multimodal treatment but remains hypothesis-generating [119].

Pemigatinib has a generally manageable toxicity profile consistent with FGFR inhibition [24,120]. Hyperphosphatemia is its characteristic on-target adverse event and results from disruption of FGF23-mediated phosphate homeostasis [117,118]. Management may include dietary modification, phosphate binders, treatment interruption, or dose adjustment [117]. Other common adverse events include stomatitis, alopecia, nail disorders, dry mouth, fatigue, diarrhea, dysgeusia, and dermatologic toxicity [24,120,121,122]. Ocular adverse events, including serous retinal and retinal pigment epithelial detachment, are less frequent but clinically important and require appropriate ophthalmologic monitoring [122]. Most toxicities can be managed with supportive care and dose modification, although chronic adverse effects may affect adherence and quality of life [120,121,122,123]. Additional reports emphasize ocular, dermatologic, and, in particular, nail monitoring issues during prolonged treatment [124,125,126], while rare inflammatory dermatologic events, including pemigatinib-associated psoriasis, have also been reported [127].

Real-world and expanded-access studies have generally supported the feasibility and activity of pemigatinib outside registration trials [128,129]. These studies include more heterogeneous populations, including patients with comorbidities, poorer performance status, previous locoregional treatment, or atypical disease courses [128]. Interpretation is limited by retrospective design, selection bias, heterogeneous molecular testing, and variable response assessment [129]. Nevertheless, available evidence is broadly consistent with the clinical activity observed in FIGHT-202 [128,129]. Canadian patient-support-program and U.S. utilization data have provided additional information on treatment patterns and outcomes in routine practice [130,131], while economic analyses highlight the influence of reimbursement, molecular-testing availability, and resource allocation on implementation [132,133].

Molecular characterization is required to identify patients with FGFR2 fusions or rearrangements who may benefit from FGFR-directed therapy [80,85,134]. However, limited tissue availability, testing infrastructure, reimbursement, and access to targeted agents remain important barriers [21]. Emerging experimental platforms, including patient-derived organoids and single-cell transcriptomics, may improve biological classification and prediction of treatment sensitivity [135], while investigation of pathways such as the FGF1–FGFR2 axis continues to refine understanding of FGFR-dependent oncogenesis in intrahepatic CCA [136].

Pemigatinib is a clinically validated targeted therapy for FGFR2 fusion/rearrangement-positive advanced CCA, supported by phase II evidence in previously treated disease and randomized phase III evidence in the first-line setting [24,89,93]. Exposure–response analyses have further characterized relationships between pemigatinib exposure, efficacy, and treatment-related toxicity [137]. Acquired resistance and optimal treatment sequencing remain important challenges [98,99,100,101,102]. The development of highly selective FGFR2 inhibitors such as lirafugratinib further illustrates the shift toward resistance-informed and molecularly guided sequential FGFR therapy [111,112,113]. Next-generation and resistance-directed agents, including the multikinase inhibitor tinengotinib, may provide additional therapeutic options following or beyond conventional FGFR inhibition [138,139].

Table 1 summarizes clinically relevant adverse events associated with pemigatinib and principal management considerations, while Table 2 outlines the major pivotal clinical trials and clinical evidence supportive of the use of pemigatinib in CCA.

## 3. Zanidatamab

Zanidatamab is a humanized bispecific HER2-directed antibody that binds extracellular domains 2 and 4 of HER2, thereby promoting receptor clustering and internalization, enhancing immune-mediated cytotoxicity, and suppressing HER2-dependent signaling [142,143,144,145]. This biparatopic configuration distinguishes zanidatamab from conventional monoclonal antibodies directed against a single HER2 epitope and may enhance receptor downregulation and antitumor activity in heterogeneous HER2-expressing tumors [143,144,145,146]. Its development extends HER2-directed therapy beyond breast and gastric cancers into BTCs, where HER2 amplification and overexpression represent therapeutically relevant alterations in selected patients [30,41,147,148]. Broader clinical development across HER2-positive solid tumors also reflects the expanding role of bispecific antibodies in targeted oncology [149,150,151].

HER2 dysregulation is observed most frequently in extrahepatic CCA and gallbladder carcinoma, with lower prevalence in intrahepatic CCA [41,148]. Depending on anatomical subtype and testing methodology, HER2 amplification or overexpression occurs in approximately 5–20% of BTCs [30,41,147,148]. HER2 activation stimulates PI3K/AKT/mTOR and RAS/RAF/MEK/ERK signaling, promoting tumor proliferation, survival, angiogenesis, and metastatic progression [152,153]. Earlier HER2-directed studies in BTC produced variable results, in part because of tumor heterogeneity, inconsistent biomarker selection, and the absence of standardized BTC-specific HER2 testing criteria [3,80]. Subsequent genomic and translational evidence has strengthened the rationale for HER2-directed treatment in appropriately selected patients [40,154,155,156,157]. Figure 3 depicts the mechanism of action of zanidatamab.

Early clinical development involved phase I dose-escalation and expansion studies in patients with advanced HER2-expressing or HER2-amplified solid tumors refractory to standard therapy [45]. These studies demonstrated preliminary antitumor activity across several HER2-driven malignancies, including BTC, and established an acceptable safety profile [43,45]. Pharmacokinetic analyses supported dose selection for subsequent development [159]. Activity across different levels of HER2 expression and amplification also suggested that dual-domain targeting might overcome selected limitations of conventional HER2-directed monoclonal antibodies [160]. However, these findings require cautious interpretation because of heterogeneous tumor populations, variable prior therapy, and small BTC-specific cohorts [43,45].

The strongest clinical evidence for zanidatamab in BTC emerged from the multicenter, open-label phase IIb HERIZON-BTC-01 trial (NCT04466891), which evaluated zanidatamab monotherapy in previously treated HER2-positive advanced BTC [46,161]. At final follow-up, zanidatamab achieved a confirmed ORR of 41.3%, an mDOR of 14.9 months, an mPFS of 5.5 months, and an mOS of 15.5 months [162]. Clinical activity was strongly associated with HER2 expression: among patients with HER2 IHC 3+ tumors, the confirmed ORR was 51.6% and median OS was 18.1 months, whereas substantially lower activity was observed in the IHC 2+ subgroup [162]. These findings supported regulatory approval and established zanidatamab as an important HER2-directed treatment option for previously treated HER2-positive BTC [46,161,162,163,164,165], a setting in which conventional therapies historically provide limited benefit [13,166].

HERIZON-BTC-01 nevertheless had important limitations. Its single-arm design precluded direct comparison with chemotherapy or other HER2-directed regimens [46,161,163]. Moreover, HER2-positive BTC encompasses anatomically and biologically heterogeneous tumors with variable HER2 expression and amplification [41,147,148], while its relative rarity limits prospective cohort sizes and the statistical power of subgroup analyses [167]. The marked difference in activity between IHC 3+ and IHC 2+ disease further emphasizes the importance of biomarker definition and appropriate patient selection [162]. Accordingly, extrapolation across all CCA subtypes should remain cautious, although evidence from multiple HER2-directed studies supports HER2 as a clinically relevant therapeutic target in biomarker-selected BTC [3,40,154,155].

The rationale for zanidatamab also builds on earlier HER2-targeted experience in BTC. In the MyPathway basket study, dual HER2 blockade with pertuzumab, a monoclonal antibody targeting HER2 dimerization, and trastuzumab, a monoclonal antibody directed against extracellular domain 4 of HER2, demonstrated clinically meaningful activity in HER2-positive metastatic BTC [44]. Antibody–drug conjugates such as trastuzumab deruxtecan, which combines HER2-directed targeting with intracellular delivery of a topoisomerase I inhibitor, have also shown activity in selected HER2-positive BTCs [168,169]. Zanidatamab differs mechanistically through simultaneous engagement of two HER2 epitopes, promoting receptor clustering, internalization, and immune-mediated antitumor effects [143,144,145]. These strategies reflect broader efforts to address HER2 heterogeneity and acquired resistance [170,171,172]. However, cross-trial comparisons are limited by differences in biomarker definitions, patient populations, previous therapy, and endpoint assessment, and no definitive evidence currently establishes the superiority of zanidatamab over other HER2-directed approaches in BTC [173,174].

The ongoing randomized phase III HERIZON-BTC-302 trial (NCT06282575) is evaluating zanidatamab in combination with gemcitabine and cisplatin, with or without immune checkpoint inhibition, as first-line therapy for HER2-positive advanced BTC [175,176]. Immune checkpoint inhibitors enhance antitumor immunity by blocking inhibitory pathways that suppress T-cell function. Experience in HER2-positive gastroesophageal adenocarcinoma supports the feasibility of combining zanidatamab with chemotherapy and immune checkpoint blockade, although disease-specific validation in BTC remains necessary [177,178,179]. The first-line development of zanidatamab may clarify its optimal positioning relative to contemporary systemic therapy, while questions concerning sequencing, resistance, cumulative toxicity, and attribution of efficacy within multidrug regimens remain unresolved [63,96].

Resistance to HER2-directed therapy in BTC remains incompletely characterized and probably involves both intrinsic and acquired mechanisms [180]. Proposed mechanisms include intratumoral HER2 heterogeneity, co-occurring genomic alterations, activation of alternative receptor tyrosine kinases, downstream pathway reactivation, epithelial–mesenchymal transition, and therapy-driven clonal selection [180,181,182,183]. Differences in HER2 expression between primary and metastatic lesions may further influence treatment sensitivity [183]. CtDNA may eventually facilitate longitudinal assessment of HER2 amplification and emerging resistance [101,184], while patient-derived xenograft models provide translational platforms for investigating zanidatamab sensitivity and resistance [185].

The TME may further influence the development and maintenance of resistance to HER2-directed therapy. In addition to tumor-intrinsic mechanisms, spatial HER2 heterogeneity and variability in receptor expression may contribute to differential sensitivity and the selection of tumor-cell populations with reduced dependence on HER2 signaling during treatment [186,187]. This may be particularly relevant to zanidatamab because its antitumor activity extends beyond dual HER2 engagement, receptor clustering, internalization, and signaling inhibition to Fc-mediated immune mechanisms, including antibody-dependent cellular cytotoxicity (ADCC), antibody-dependent cellular phagocytosis (ADCP), and complement-dependent cytotoxicity (CDC) [146]. Accordingly, changes in HER2 expression and in the composition or functional state of immune components within the TME could potentially influence both target-dependent and immune-mediated components of zanidatamab activity [146,186,187]. Although the specific contribution of TME remodeling to acquired zanidatamab resistance in CCA remains incompletely defined, these observations support considering resistance as an interaction between tumor-intrinsic HER2 evolution and the surrounding immune microenvironment.

Zanidatamab is generally associated with a manageable toxicity profile consistent with HER2-directed antibody therapy [46,163,184]. Diarrhea and infusion-related reactions are among the most frequently reported adverse events and are generally manageable with supportive treatment, premedication, and infusion-rate modification when appropriate [46,159,180]. Other adverse events include fatigue, nausea, abdominal discomfort, rash, decreased appetite, and transient liver-function abnormalities [46,163,184]. Cardiac dysfunction appears relatively uncommon, although monitoring remains appropriate given the cardiotoxic potential of some HER2-targeted therapies [188]. Safety findings across breast, gastric/gastroesophageal, endometrial, and salivary gland cancers have likewise supported a generally manageable toxicity profile [189,190,191,192].

Real-world and expanded-access experience has further supported the feasibility of zanidatamab outside controlled clinical trials [163]. Such populations may include patients with comorbidities, impaired performance status, previous locoregional treatment, and other characteristics underrepresented in registration studies [163]. Interpretation remains limited by retrospective design, variable HER2 testing, heterogeneous treatment sequencing, and inconsistent radiologic assessment [129]. Nevertheless, available observational findings are broadly consistent with prospective clinical experience [129,163].

Implementation of HER2-directed treatment depends on reliable identification of HER2-positive disease and consistent interpretation of biomarker results [11,34,80,134,193]. Important barriers include variability in testing methodology, limited tissue availability, access to specialized diagnostics, reimbursement, and availability of targeted therapies [21]. Patient-derived models and other translational platforms may further refine treatment selection as HER2-directed options expand [157].

Zanidatamab is a clinically validated HER2-directed therapy for previously treated HER2-positive advanced BTC, with final HERIZON-BTC-01 results demonstrating substantial and durable antitumor activity [46,161,162,163]. The greater benefit observed in IHC 3+ disease underscores the importance of HER2 expression level for patient selection [164]. Unresolved issues include optimal biomarker thresholds, acquired resistance, treatment sequencing, comparative efficacy relative to other HER2-directed agents, and integration with first-line chemotherapy and immunotherapy [63,174,175,176,194]. Continued development across other HER2-driven malignancies may provide additional biological and clinical insights relevant to BTC [195,196,197,198,199], while improved characterization of HER2 heterogeneity and resistance should help refine future therapeutic strategies [99,101,180,181,182,183]. Case-level observations of long-term survival in CCA further illustrate the potential contribution of integrated systemic, locoregional, and multidisciplinary treatment [200].

Table 3 summarizes clinically relevant adverse events associated with zanidatamab and principal management considerations, while Table 4 outlines the major pivotal clinical trials and clinical evidence supportive of the use zanidatamab in CCA.

## 4. Ivosidenib

Ivosidenib is an orally administered, first-in-class small-molecule inhibitor of IDH1m that selectively suppresses the neomorphic enzymatic activity responsible for converting α-ketoglutarate into the oncometabolite D-2-hydroxyglutarate [201,202,203,204]. IDH1 mutations occur predominantly in intrahepatic CCA, affecting approximately 10–20% of cases and defining a biologically distinct molecular subgroup [21,48,205,206]. Accumulation of D-2-hydroxyglutarate promotes epigenetic dysregulation, impaired cellular differentiation, metabolic reprogramming, and tumorigenic plasticity, providing a strong rationale for selective IDH1 inhibition [49,202,204,207]. Broader reviews of IDH-targeted oncology similarly identify ivosidenib as a representative example of therapeutically targeting mutant metabolic enzymes [208,209,210]. Figure 4 illustrates its mechanism of action.

Initial clinical evidence arose from a phase I dose-escalation and expansion study in patients with advanced IDH1-mutant solid tumors, including a dedicated CCA cohort [52]. Ivosidenib demonstrated acceptable tolerability and durable disease stabilization, with relatively few objective responses [52,212]. This pattern is consistent with a predominantly cytostatic effect in CCA, making PFS and sustained disease control more informative indicators of activity than radiographic tumor shrinkage alone [52,212,213]. Although the study was non-randomized and involved a molecularly selected population, it provided the basis for subsequent phase III evaluation [52]. Later reviews have similarly emphasized disease control rather than response rate as the principal manifestation of clinical activity [214,215,216,217].

The pivotal randomized, double-blind, placebo-controlled phase III ClarIDHy trial (AG120-C-005; NCT02989857) evaluated ivosidenib in patients with unresectable or metastatic IDH1-mutant CCA after progression on prior systemic therapy [50,218]. MPFS was 2.7 months with ivosidenib compared with 1.4 months with placebo (HR, 0.37; 95% CI, 0.25–0.54), while the ORR with ivosidenib was 2.4%, consistent with a predominantly disease-stabilizing rather than tumor-shrinking effect [50]. In the final analysis, mOS was 10.3 months with ivosidenib and 7.5 months with placebo (HR, 0.79; 95% CI, 0.56–1.12) [51]. Interpretation of OS was substantially affected by crossover, as 70% of placebo-treated patients subsequently received ivosidenib; after adjustment for crossover, estimated median OS in the placebo group was 5.1 months (HR, 0.49; 95% CI, 0.34–0.70) [51]. These findings established randomized phase III evidence supporting IDH1-directed therapy in previously treated IDH1-mutant CCA [50,51,218,219,220,221].

Health-related quality-of-life analyses from ClarIDHy additionally supported preservation of physical functioning and symptom control [51,222]. This is clinically relevant in advanced CCA, where biliary obstruction, hepatic dysfunction, fatigue, cachexia, and treatment toxicity may substantially impair quality of life [223,224]. An orally administered therapy capable of delaying progression without substantial cumulative toxicity may therefore be particularly valuable after gemcitabine- and platinum-based treatment [50,51,217,222,223,224]. Nevertheless, ivosidenib should not be regarded as a rapid cytoreductive therapy, and its clinical benefit should be interpreted primarily in terms of delayed progression and sustained disease control [50,51,218]. Reviews across CCA and acute myeloid leukemia (AML) similarly describe a favorable therapeutic index while emphasizing appropriate molecular selection and clinical monitoring [225].

Compared with standard second-line chemotherapy, ivosidenib occupies a distinct therapeutic niche. The ABC-06 trial established FOLFOX, a cytotoxic regimen combining folinic acid, fluorouracil, and oxaliplatin, as a second-line option after gemcitabine–cisplatin, but the survival gain was modest and accompanied by conventional chemotherapy-associated toxicity [13]. In IDH1-mutant CCA, ivosidenib provides a biomarker-directed alternative with a generally favorable tolerability profile [50,51,218,226]. Direct comparison with chemotherapy is not possible because ClarIDHy used placebo rather than an active comparator [50,218]. Treatment selection after first-line therapy should therefore consider molecular status, disease tempo, symptom burden, hepatic function, performance status, previous toxicities, and patient preference [3,63,80,134,223]. Economic analyses further indicate that the practical value of ivosidenib is influenced by testing infrastructure, reimbursement, treatment sequencing, and healthcare context [227,228].

Real-world and expanded-access studies have supported the feasibility of ivosidenib beyond randomized trials [229,230,231]. The phase IIIb ProvIDHe study was designed to further characterize safety and effectiveness in a broader population of previously treated IDH1-mutant CCA, with preliminary findings broadly consistent with the pivotal development program [229]. Multicenter and Italian real-world analyses have likewise reported clinically relevant disease control and complementary survival data in previously treated metastatic IDH1-mutant intrahepatic CCA [230,232]. These studies extend evidence to more heterogeneous populations but remain limited by non-randomized design, selection bias, inconsistent imaging schedules, and incomplete documentation [229,230,231,232].

The adverse-event profile of ivosidenib is generally manageable [50,51,218,226]. Common treatment-emergent adverse events include fatigue, nausea, abdominal pain, diarrhea, decreased appetite, ascites, anemia, and peripheral edema, several of which overlap with manifestations of advanced CCA [50,51,218,226]. QT interval prolongation is a clinically important toxicity requiring baseline and periodic electrocardiographic monitoring, correction of electrolyte disturbances, and caution with concomitant QT-prolonging medications [226]. Differentiation syndrome, a systemic inflammatory complication of IDH inhibition well recognized in hematologic malignancies, is a labeled risk but appears rare in solid tumors such as CCA [226,233]. Liver-function abnormalities require careful interpretation because they may reflect treatment toxicity, biliary obstruction, cholangitis, hepatic progression, or previous locoregional therapy [223,224,226]. Taken together, systematic safety analyses support a favorable tolerability profile while reinforcing the need for routine clinical monitoring [221].

Resistance to IDH1 inhibition remains an important biological challenge. Reported and proposed mechanisms include second-site IDH1 mutations, isoform switching to IDH2 mutations, restoration of 2-hydroxyglutarate production, compensatory oncogenic signaling, and clonal evolution under therapeutic pressure [234,235,236,237]. In CCA, secondary IDH1 mutations and emergent oncogenic IDH2 alterations have been directly implicated in acquired resistance to ivosidenib [238].

In addition to secondary IDH1 mutations, acquired resistance to ivosidenib may occur through IDH isoform switching involving the acquisition of an oncogenic IDH2 mutation. Clinical observations in IDH1-mutant CCA have demonstrated the emergence of mutant IDH2 during ivosidenib treatment, restoring 2-hydroxyglutarate (2-HG) production despite pharmacological inhibition of IDH1m [235,238]. This finding suggests continued dependence of resistant tumors on the 2-HG-driven oncogenic phenotype and provides a biological rationale for targeting mutant IDH2 after molecularly confirmed isoform switching [235,238]. Enasidenib is an oral, selective mutant IDH2 inhibitor with established clinical activity in IDH2-mutated acute myeloid leukemia [239]. However, its efficacy in IDH2-mutant CCA, particularly in acquired IDH2-mediated resistance to ivosidenib, remains unestablished. In the reported CCA case with acquired IDH2 R172V following ivosidenib treatment, subsequent enasidenib therapy did not result in documented clinical benefit [235]. Consequently, sequential mutant IDH inhibition, combined IDH1/IDH2 inhibition, or dual IDH1/IDH2 inhibitors represent biologically plausible but currently investigational strategies rather than established treatments for ivosidenib-resistant CCA [235,238]. Re-biopsy and/or ctDNA analysis at disease progression may therefore be valuable for identifying IDH isoform switching, secondary IDH1 mutations, or alternative resistance mechanisms and could inform future genotype-directed therapeutic strategies [235,238].

The interplay between IDH1 signaling, therapeutic resistance, and the TME may be particularly relevant in IDH1-mutant CCA. Beyond the tumor-intrinsic resistance mechanisms described above, mutant-IDH1-associated metabolic reprogramming has been linked to an immunosuppressive microenvironment. IDH1-mutant CCA has been associated with reduced CD8+ T-cell infiltration and increased M2-like tumor-associated macrophage infiltration, with tumor-derived CCL2 implicated in macrophage recruitment and immunosuppression [240]. Moreover, MAPK pathway alterations emerging during mutant-IDH1 inhibition may attenuate interferon-responsive transcriptional programs, providing a mechanistic link between tumor-cell-intrinsic resistance and impaired antitumor immune signaling [241]. These findings suggest that resistance to ivosidenib may involve reciprocal interactions among clonal evolution, oncogenic signaling, metabolic adaptation, and immune remodeling rather than restoration of tumor-intrinsic oncogenic signaling alone [240,241]. More broadly, ctDNA may facilitate longitudinal detection of these and other emerging resistance alterations [101,242], although ctDNA-guided treatment adaptation remains investigational and does not replace validated tissue-based confirmation for initial treatment selection [101,173,242]. Emerging ctDNA approaches for molecular residual disease assessment may further refine monitoring in IDH-mutant CCA [243].

Combination strategies involving ivosidenib remain investigational. Potential approaches include combinations with chemotherapy, immune checkpoint inhibitors, or other targeted agents [63,173,244,245]. Immune checkpoint inhibitors enhance antitumor T-cell activity by blocking inhibitory immune signaling, and the immunosuppressive features associated with IDH1-mutant CCA described above provide a biological rationale for investigating their combination with IDH1 inhibition [201,244,245]. Early phase I studies combining gemcitabine and cisplatin with ivosidenib or pemigatinib provide preliminary evidence for integrating targeted therapy with chemotherapy, but these regimens remain investigational [97]. Preclinical studies have also identified mitochondrial dependencies and potential sensitivity to MDM2 inhibition, which targets a negative regulator of p53, in IDH1-mutant intrahepatic CCA [246,247]. Prospective evidence remains insufficient to support routine combination therapy, and ivosidenib monotherapy remains the best-supported approach for previously treated advanced IDH1-mutant CCA [50,51,173,218,226,244,245].

The therapeutic role of ivosidenib depends on reliable identification of IDH1-mutant disease [3,63,80,134]. Its use is most appropriate after progression on standard systemic therapy when disease behavior permits a disease-stabilizing rather than rapidly cytoreductive strategy [50,51,218]. Molecular context may also influence sensitivity to conventional therapy; recent analyses examining IDH1 R132 mutations, HER2 positivity, and platinum-based treatment suggest potential relationships that warrant further investigation [248].

Ivosidenib is a clinically validated targeted therapy for previously treated IDH1-mutant advanced CCA, with randomized evidence demonstrating delayed disease progression despite a low ORR [50,51,218]. Its generally favorable tolerability further supports its use in this molecularly selected population [50,51,218,226]. Important unresolved issues include acquired resistance, optimal treatment sequencing, and the role of combination strategies [13,50,51,215]. Future development will likely focus on resistance characterization, longitudinal molecular monitoring, and prospective evaluation of biologically rational combinations [173,237,242,244,245]. Emerging CCA targets beyond FGFR2 and IDH1 may further expand molecularly directed treatment options [249,250,251], while preliminary evidence that ivosidenib may modify chemoresistance in selected wild-type IDH1 intrahepatic CCA models remains investigational and does not alter its current biomarker-defined clinical use [252].

Table 5 summarizes clinically relevant adverse events associated with ivosidenib and principal management considerations, while Table 6 outlines the major pivotal clinical trials and clinical evidence supportive of the use of ivosidenib in CCA.

## 5. Future Directions in Molecularly Guided Therapy and Tumor Microenvironment-Targeted Strategies for Cholangiocarcinoma

The therapeutic landscape of CCA is evolving rapidly as molecular profiling and biomarker-directed therapies increasingly complement conventional anatomical classification. Actionable alterations include FGFR2 fusions or rearrangements, IDH1 mutations, HER2 amplification or overexpression, BRAF V600E mutations, MSI-H/dMMR status, KRAS G12C alterations, and NTRK fusions [20,48,80,134]. Recent analyses of intrahepatic CCA further support integration of genomic, radiologic, and histopathological features for treatment stratification [258,259]. The clinical development of pemigatinib, zanidatamab, and ivosidenib illustrates the therapeutic value of matching molecular drivers with targeted interventions and extends earlier predictions that molecularly guided treatment would become central to CCA management [24,46,260,261,262,263].

A major priority is timely and equitable access to molecular testing. Current evidence supports NGS early in the course of advanced disease to identify actionable alterations, facilitate treatment sequencing, and improve eligibility for biomarker-selected clinical trials [80,134,173]. Predictive immunohistochemistry remains useful for selected biomarkers, although standardized interpretation and integration with molecular assays are required [264]. Important barriers include limited tissue availability, reimbursement constraints, restricted access to NGS platforms, prolonged turnaround times, and insufficient molecular tumor-board infrastructure [21,63,223]. Future progress will therefore depend not only on development of new therapies but also on broader access to reliable molecular diagnostics.

Liquid biopsy and ctDNA analysis may further improve molecular monitoring in CCA [99,101,265]. Potential applications include detection of actionable alterations, assessment of treatment response, identification of minimal residual disease, and recognition of resistance-associated molecular changes [99,101,265]. This is particularly relevant when tissue acquisition is difficult because of anatomical location, low tumor cellularity, previous biliary intervention, or advanced disease [2]. Serial ctDNA analysis could eventually identify resistance mechanisms such as secondary FGFR2 mutations, evolving HER2-pathway alterations, or clonal changes during IDH1 inhibition [2,27,99,101,265]. However, ctDNA-guided treatment adaptation remains investigational and requires prospective validation and assay standardization before routine implementation [266].

Therapeutic sequencing and resistance-directed treatment represent additional major challenges. Acquired resistance is common with targeted therapies and may arise through secondary kinase-domain mutations, bypass signaling, receptor heterogeneity, downstream pathway activation, metabolic reprogramming, and clonal evolution [98,102,235,267,268]. In FGFR2-rearranged CCA, secondary FGFR2 mutations and alternative signaling pathways are well-established resistance mechanisms [98,102,244], while HER2-directed therapy may be affected by heterogeneous receptor expression and compensatory signaling [267,268]. Resistance to IDH1m inhibition can involve restoration of oncogenic metabolic signaling or clonal evolution [234]. Future therapeutic strategies may therefore require integration of tumor-intrinsic molecular targeting with approaches directed against adaptive signaling and tumor microenvironment-associated mechanisms, including MET-, AXL-, and TGF-β-related pathways implicated in epithelial–mesenchymal plasticity, invasion, and therapeutic resistance [269]. These mechanisms provide the rationale for next-generation inhibitors, sequential targeted therapy, and combinations selected according to specific resistance pathways [270]. Potential strategies include targeted agents combined with chemotherapy, immune checkpoint inhibitors, antiangiogenic therapies, antibody–drug conjugates, or other pathway-directed treatments [14,112,243,271]. Comparative systematic reviews and network meta-analyses may help contextualize these approaches, although heterogeneity among trials limits direct comparisons [272].

Immunotherapy also remains an important area of investigation. Durvalumab, an anti-PD-L1 immune checkpoint inhibitor, combined with gemcitabine and cisplatin established chemoimmunotherapy as a first-line standard for advanced BTC, although benefit varies among molecular and immunological subgroups [113]. FGFR2-rearranged tumors may display relatively immune-excluded microenvironments, potentially limiting sensitivity to immune checkpoint inhibition alone [273]. Conversely, specific genomic and epigenetic contexts may increase tumor immunogenicity and provide a rationale for targeted–immunotherapy combinations [274]. Spatial profiling has identified distinct immune phenotypes across BTC that are partly independent of anatomical origin, suggesting that immune classification may eventually complement genomic stratification [275]. Future trials will therefore benefit from integrated analysis of immune contexture, ctDNA, resistance biomarkers, and the tumor microenvironment [116,117,271,273,274]. Cancer-associated fibroblasts, stromal components, and immunosuppressive cellular networks are particularly relevant therapeutic targets in intrahepatic CCA [276,277].

Artificial intelligence (AI), machine learning, and computational oncology may further refine CCA management [31,32]. Potential applications include radiographic interpretation, digital pathology, biomarker prediction, prognostic stratification, treatment selection, and identification of resistance patterns from integrated clinical and molecular datasets [278,279]. Radiomic and molecular-signature approaches may also enable non-invasive prediction of biologically relevant tumor features [258]. These technologies remain exploratory, however, and require rigorous validation, standardized datasets, transparent algorithms, appropriate regulatory oversight, and careful consideration of ethical and interpretability issues before clinical implementation [278,279,280].

Molecular classification itself is also likely to become more refined. Conventional division into intrahepatic, perihilar, and distal CCA is increasingly complemented by biologically defined subgroups characterized by specific genomic alterations, signaling dependencies, immune phenotypes, and therapeutic vulnerabilities [20,48,56]. Such classification may eventually support treatment algorithms driven more strongly by tumor biology than by anatomical origin alone [56]. Beyond established oncogenic drivers, emerging research implicates bile acid metabolism, gut microbiome interactions, peroxisome-associated pathways, and metabolic–inflammatory crosstalk in cholangiocarcinogenesis and therapeutic vulnerability [281,282,283].

The development of next-generation targeted agents, bispecific antibodies, and antibody–drug conjugates is expected to further expand available treatment options. Antibody–drug conjugates combine tumor-specific antibody recognition with intracellular delivery of a cytotoxic payload, whereas bispecific antibodies can engage two epitopes or molecular targets simultaneously. HER2-directed antibody–drug conjugates, irreversible FGFR inhibitors, KRAS G12C inhibitors, and novel metabolic pathway inhibitors are under investigation in BTC [26,267,284,285]. Retrospective experience across gastrointestinal malignancies supports activity of several HER2-directed strategies and highlights the need to determine optimal sequencing among monoclonal antibodies, bispecific antibodies, and antibody–drug conjugates [286]. Bispecific immune-engaging antibodies and cellular therapies may further broaden therapeutic options [287], while emerging delivery platforms such as lipid nanoparticles and extracellular vesicles are being explored for targeted drug delivery and immune modulation [288]. Their clinical role remains uncertain and will depend on efficacy, toxicity, patient selection, and resistance patterns.

Health-economic considerations will become increasingly important as precision therapies expand. Pemigatinib, zanidatamab, and ivosidenib provide clinically meaningful benefit in selected populations but are associated with substantial drug-acquisition and molecular-testing costs [289,290,291]. Economic evaluation is complicated by small biomarker-defined trial populations, limited long-term outcome data, crossover, differences in reimbursement systems, and the additional costs of repeated molecular testing and chronic toxicity management [289,290,291]. Conversely, biomarker-guided treatment may reduce exposure to ineffective therapy, decrease avoidable toxicity, and improve treatment allocation and quality-adjusted survival [289,290,291,292]. Future cost-effectiveness analyses should therefore incorporate real-world effectiveness, patient-reported outcomes, diagnostic costs, and longer-term resource utilization [290,291,292].

Clinical-trial design will also need to evolve as therapeutically relevant subgroups become smaller. Basket trials, which enroll patients according to a shared molecular alteration across tumor types, umbrella trials, which assign treatments according to molecular subgroups within one cancer type, and adaptive master protocols may improve evaluation of rare biomarker-defined populations [293]. These designs may accelerate testing of new therapies and resistance-directed strategies but introduce challenges related to small sample sizes, biomarker heterogeneity, endpoint selection, and cross-cohort comparison [293,294]. International collaboration and harmonization of molecular-testing standards will therefore remain essential.

Locoregional and perioperative strategies will continue to complement systemic treatment. Hepatic artery infusion chemotherapy and selective internal radiotherapy are being refined for selected patients with liver-dominant disease and may increasingly be incorporated into multimodal treatment strategies [295,296]. These approaches are unlikely to replace molecularly targeted systemic therapy but may improve disease control when anatomical distribution, hepatic reserve, and treatment goals support combined management.

Overall, future CCA management will depend on better integration of molecular diagnostics, resistance monitoring, rational treatment sequencing, emerging targeted and immune-based therapies, and multidisciplinary care. Pemigatinib, zanidatamab, and ivosidenib have demonstrated that biomarker-defined subgroups can derive clinically meaningful benefit from mechanism-based therapy [24,46,260,261]. The next phase of progress will require improved characterization of resistance, validation of dynamic biomarkers, efficient trial designs, broader diagnostic access, and economically sustainable implementation of precision treatment strategies.

Table 7 summarizes contemporary management of CCA, including indications, representative regimens, molecular biomarkers, toxicity considerations, and sup-porting evidence, while Figure 5 outlines diagnosis-driven therapeutic pathways and treatment algorithms integrating molecular profiling and treatment sequencing in CCA.

## 6. Conclusions

Advanced CCA has become an important model of biomarker-directed cancer therapy, with molecular alterations increasingly informing treatment selection in defined patient subgroups. The clinical development of pemigatinib, zanidatamab, and ivosidenib demonstrates that targeting FGFR2, HER2, and IDH1m can provide clinically meaningful benefit beyond conventional cytotoxic therapy and has broadened treatment options for patients with molecularly selected disease.

Despite these advances, CCA remains characterized by substantial biological heterogeneity, variable treatment sensitivity, and acquired resistance. Intratumoral diversity, clonal evolution, and differences in biomarker prevalence continue to limit the durability and generalizability of targeted treatment responses. Improving outcomes will therefore require better characterization of resistance mechanisms, more effective treatment sequencing, and rational integration of targeted agents with other therapeutic modalities.

Future progress will likely depend on validated molecular and longitudinal biomarkers, next-generation targeted therapies, antibody–drug conjugates, immunotherapeutic combinations, and biomarker-enriched clinical trials. Equally important will be wider access to reliable molecular testing and sustainable implementation of targeted treatment in routine practice.

The transition from predominantly histology-based treatment toward molecularly informed therapeutic decision-making represents a major advance in CCA management. Continued translational and prospective clinical research will be essential to convert these molecular insights into more durable and broadly accessible improvements in outcomes for patients with advanced CCA.

## Figures and Tables

**Figure 1:**
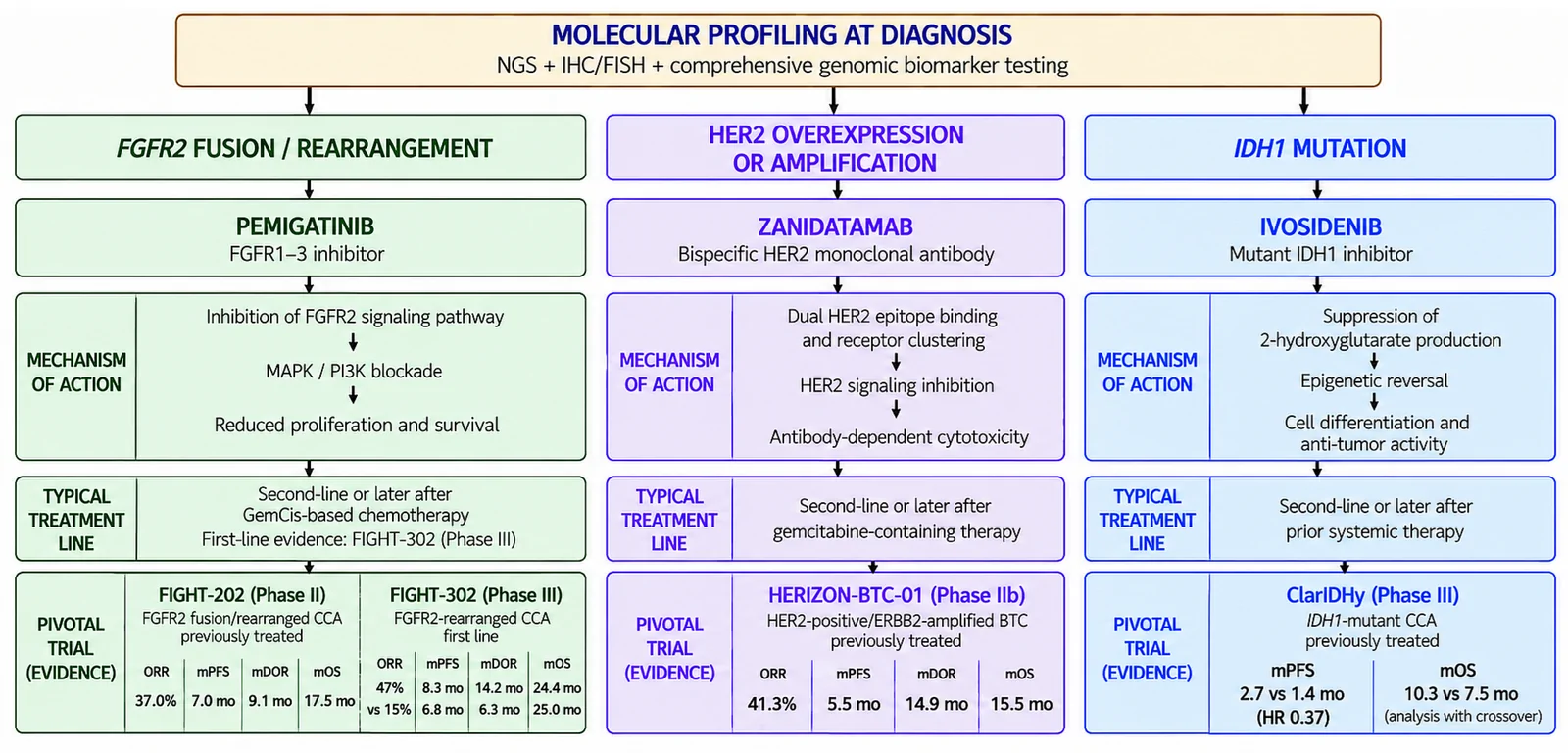
Schematic overview of molecularly guided treatment selection in advanced cholangiocarcinoma, integrating comprehensive molecular profiling and biomarker-based patient stratification with pivotal clinical trial evidence and key quantitative efficacy outcomes. The figure highlights actionable FGFR2, HER2, and IDH1 alterations and their corresponding targeted therapies—pemigatinib, zanidatamab, and ivosidenib, respectively—according to treatment setting and line of therapy. The pivotal evidence includes FIGHT-202 (Phase II), demonstrating clinically meaningful objective responses to pemigatinib in previously treated FGFR2 fusion/rearranged CCA; FIGHT-302 (Phase III), evaluating pemigatinib versus standard chemotherapy in the first-line setting; HERIZON-BTC-01 (Phase IIb), demonstrating substantial and durable responses to zanidatamab in previously treated HER2-positive/ERBB2-amplified biliary tract cancer; and ClarIDHy (Phase III), demonstrating improved PFS with ivosidenib in previously treated IDH1-mutant CCA. The magnitude and pattern of clinical benefit differ among these strategies: pemigatinib and zanidatamab are associated with substantial objective responses, whereas the benefit of ivosidenib is characterized predominantly by disease stabilization and delayed progression. Direct cross-trial comparisons should be avoided because of differences in study design, treatment line, molecular eligibility criteria, patient populations, and comparator arms. The scheme represents an original conceptual synthesis of the currently available evidence and is aligned with guideline-based principles of comprehensive molecular profiling, biomarker-directed treatment selection, and therapeutic sequencing. All abbreviations employed are defined in the Abbreviations section.

**Figure 2:**
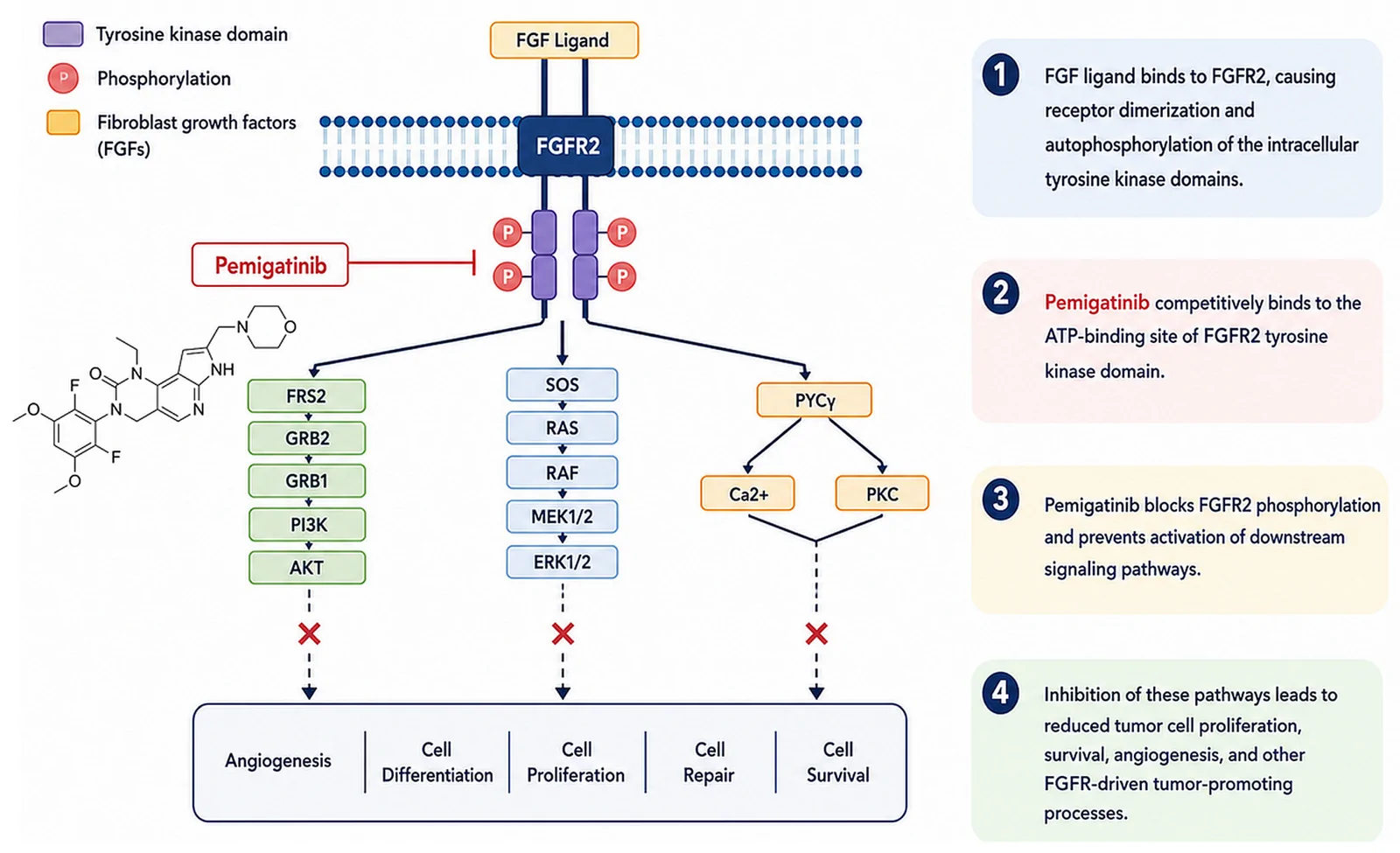
Schematic representation of the mechanism of action of pemigatinib. Original schematic illustration created by the authors based on the mechanism described in ref. [84]. FGF ligand binding to FGFR2 induces receptor dimerization and autophosphorylation of intracellular tyrosine kinase domains, resulting in activation of downstream signaling cascades, including the FRS2/PI3K/AKT, RAS/RAF/MEK/ERK, and PLCγ/PKC pathways. Pemigatinib selectively and competitively inhibits the ATP-binding site of FGFR2, thereby preventing receptor phosphorylation and downstream signal transduction. Inhibition of these oncogenic signaling pathways reduces tumor cell proliferation, survival, angiogenesis, and other FGFR-driven cellular processes. All abbreviations employed are defined in the text in the Abbreviations section.

**Figure 3:**
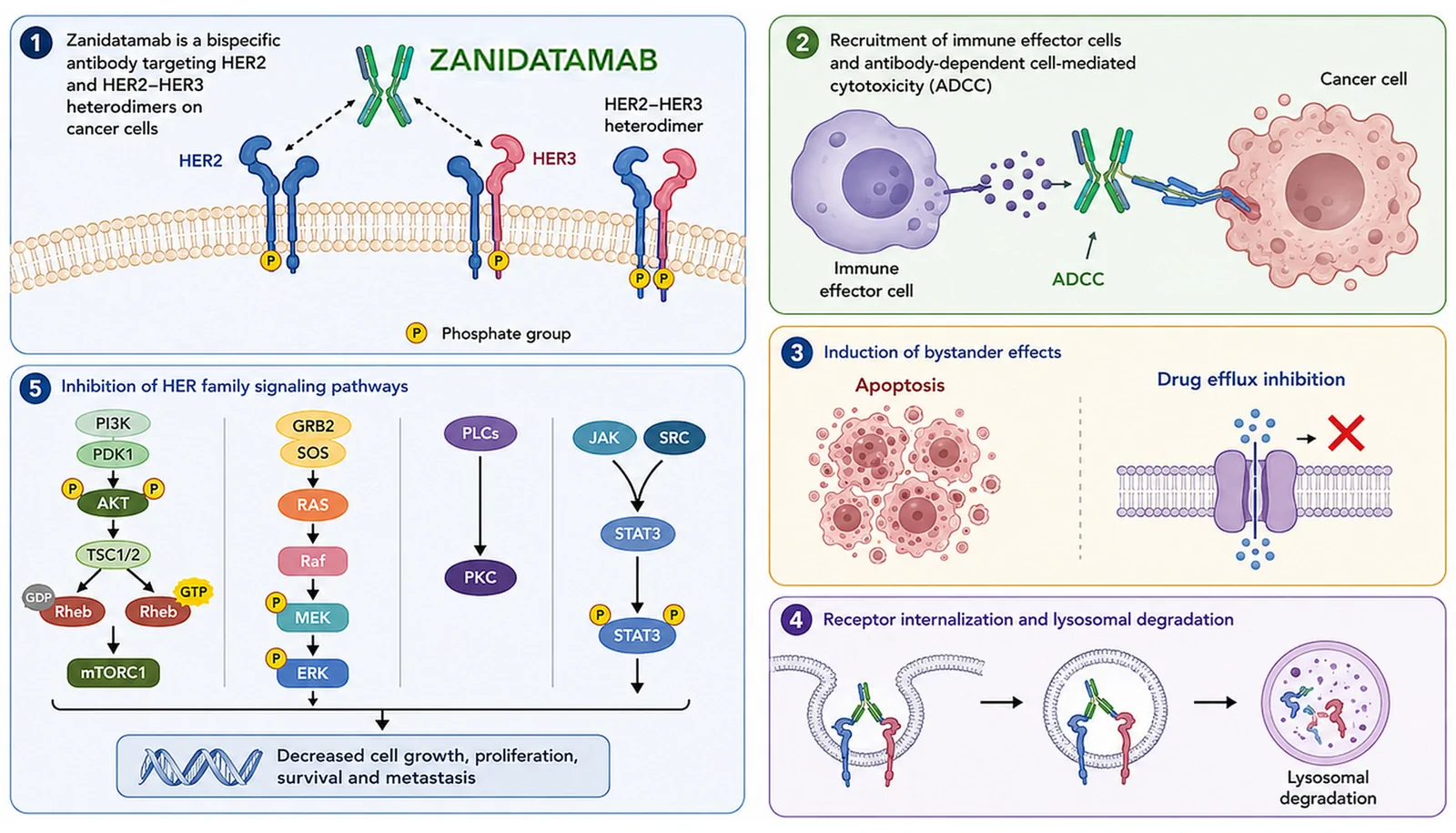
Schematic illustration of the mechanism of action of zanidatamab a bispecific HER2-targeted antibody designed to bind two distinct extracellular domains of HER2 simultaneously. Original schematic illustration created by the authors based on the mechanism described in ref. [158]. Zanidatamab promotes HER2 receptor clustering and inhibits HER2/HER3 heterodimerization, thereby suppressing downstream oncogenic signaling pathways. In addition, zanidatamab enhances immune-mediated antitumor activity through recruitment of immune effector cells and induction of ADCC. The figure further demonstrates receptor internalization and lysosomal degradation following antibody binding, as well as potential bystander antitumor effects and inhibition of drug efflux mechanisms. In combination, these processes result in suppression of HER family signaling pathways, including PI3K/AKT/mTOR, RAS/RAF/MEK/ERK, PLC/PKC, and JAK/STAT signaling cascades, ultimately leading to decreased tumor cell proliferation, survival, and metastatic potential. All abbreviations employed are defined in the text in the Abbreviations section.

**Figure 4:**
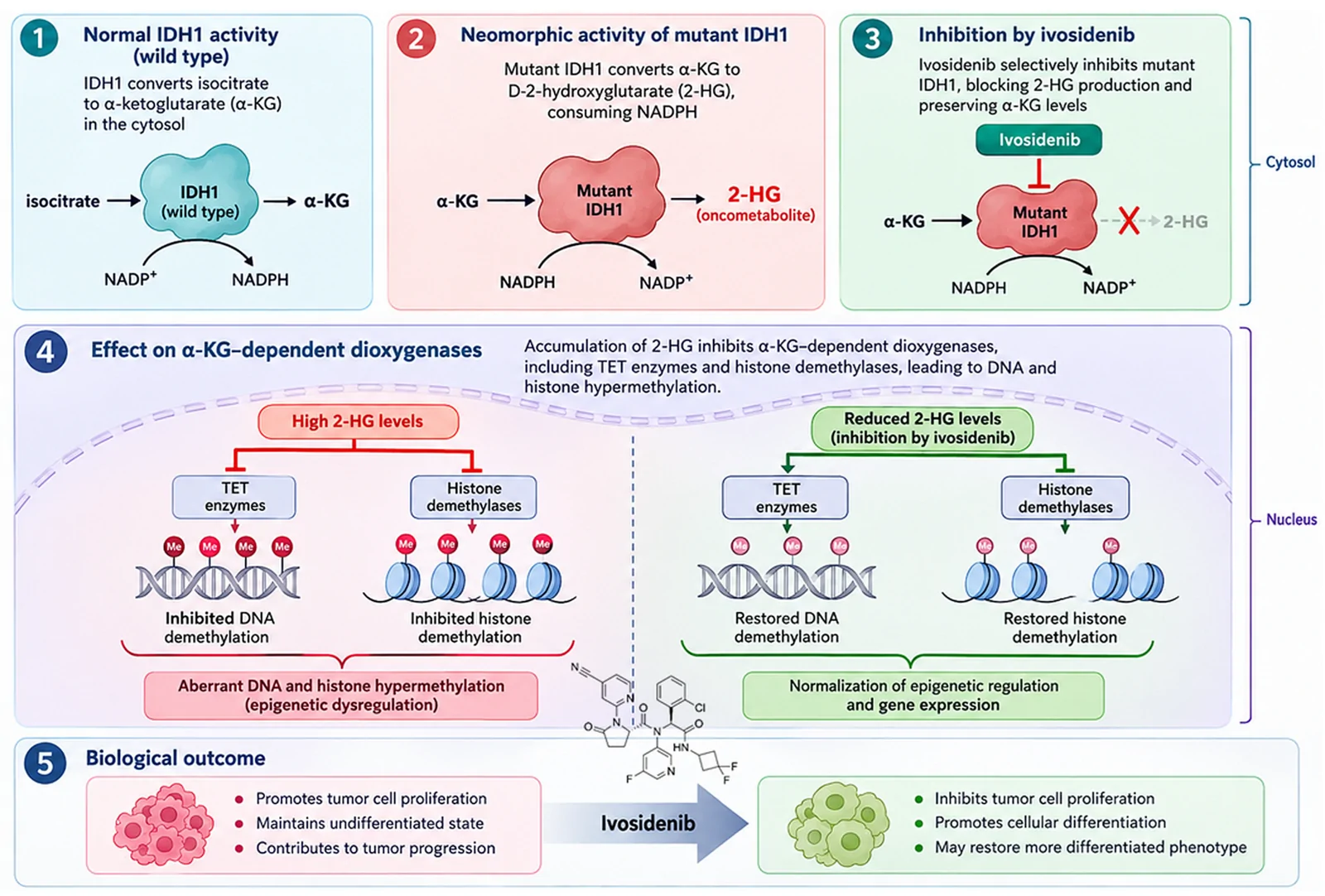
Schematic representation of the mechanism of action of ivosidenib, a selective inhibitor of mutant isocitrate dehydrogenase 1. Original schematic illustration created by the authors based on the mechanism described in ref. [211]. Under physiological conditions, wild-type IDH1 catalyzes the conversion of isocitrate to α-ketoglutarate (α-KG) with concomitant production of NADPH. In IDH1-mutant cells, the mutant enzyme acquires neomorphic activity and converts α-KG to the oncometabolite D-2-hydroxyglutarate (2-HG), consuming NADPH. Accumulation of 2-HG inhibits α-KG-dependent dioxygenases, including ten-eleven translocation (TET) enzymes and histone demethylases, resulting in aberrant DNA and histone hypermethylation and impaired cellular differentiation. Ivosidenib selectively inhibits mutant IDH1, thereby suppressing 2-HG production and preserving α-KG availability. Reduced 2-HG levels restore α-KG-dependent dioxygenase activity, facilitating DNA and histone demethylation and normalization of epigenetic regulation and gene expression. These effects promote cellular differentiation and contribute to inhibition of tumor cell proliferation. All abbreviations employed are defined in the text in the Abbreviations section.

**Figure 5:**
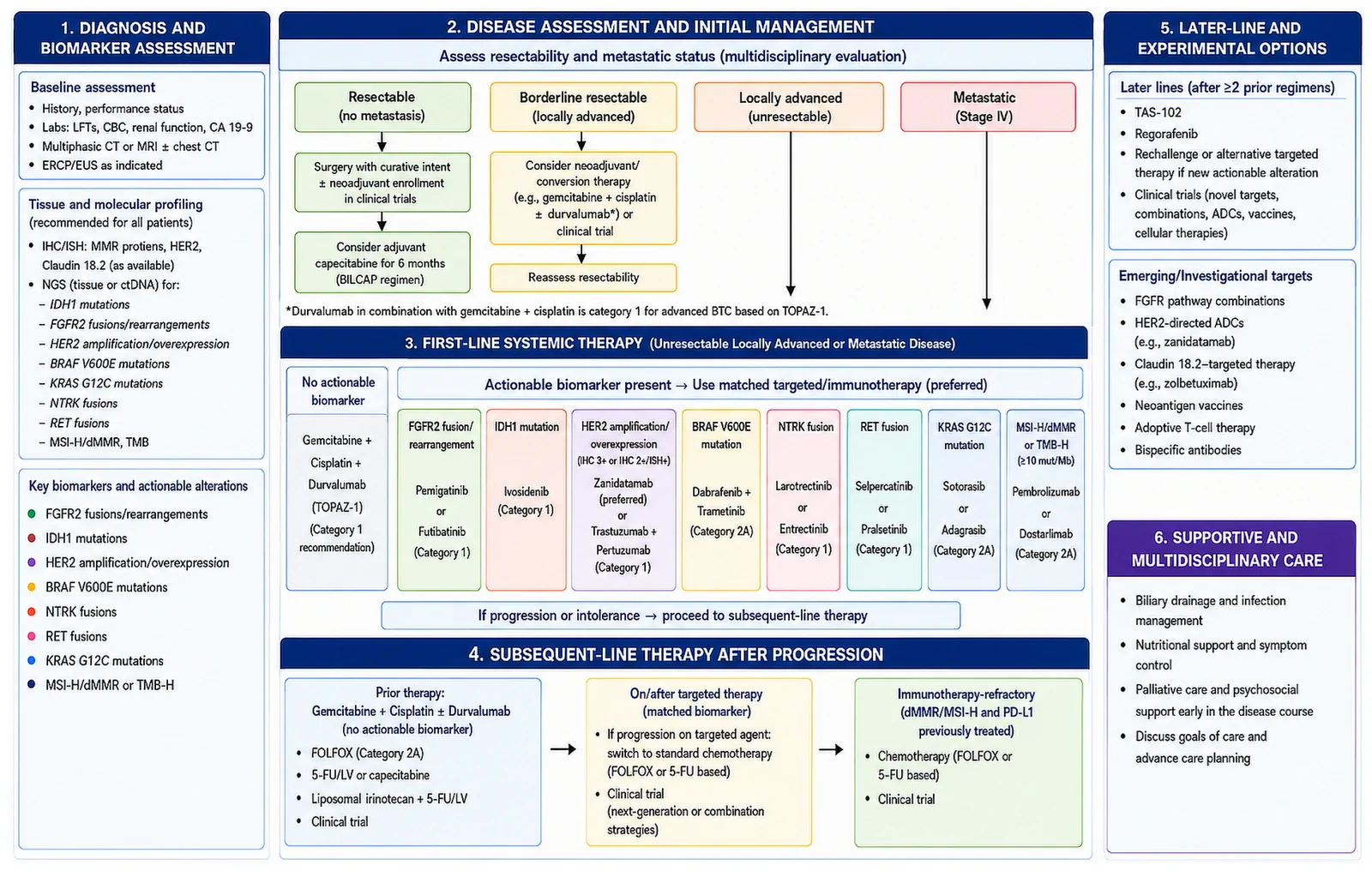
Therapeutic pathways and treatment algorithms in CCA integrating biomarker-guided treatment selection and therapeutic sequencing. The figure illustrates a diagnosis-driven management framework based on comprehensive molecular profiling, patient fitness, disease characteristics, and risk stratification. It incorporates systemic chemotherapy, immunotherapy, targeted therapies, consolidation strategies, surveillance, and relapse management in accordance with the core principles of contemporary NCCN and ESMO recommendations. The proposed treatment algorithm is intended as a literature-based conceptual overview summarizing current evidence from published clinical trials and international guidelines. It is not intended to replace individualized clinical decision-making or multidisciplinary evaluation, which should always consider patient performance status, comorbidities, treatment-related toxicities, molecular findings, physician judgment, and institutional expertise. This schematic is an original, concept-based representation and does not constitute a direct reproduction of any published guideline, figure, or external source. Selected treatment pathways, including emerging targeted combinations, maintenance approaches, and resistance-directed strategies, reflect evolving clinical evidence, current translational research, and investigational therapeutic concepts undergoing active clinical evaluation. All abbreviations employed are defined in the text in the Abbreviations section.

**Table 1:** Characteristic and clinically relevant adverse events associated with pemigatinib and principal management considerations [24,94,95,140]. All abbreviations employed are defined in the text in the Abbreviations section.

Characteristic/Clinically Relevant Toxicity	Frequency/Clinical Relevance	Principal Management Considerations
Hyperphosphatemia	Very common; characteristic on-target effect of FGFR inhibition; usually grade 1–2	Monitor serum phosphate; dietary phosphate restriction and phosphate-lowering therapy as appropriate; treatment interruption or dose reduction for persistent/severe elevations
Ocular toxicity (dry eye and retinal pigment epithelial/serous retinal detachment)	Dry eye is common; retinal toxicity is less frequent but clinically important and may affect vision	Baseline and periodic ophthalmologic assessment; prompt evaluation of visual symptoms; treatment interruption or dose modification for clinically significant retinal toxicity
Nail and dermatologic toxicities	Common; usually low grade; includes onycholysis, nail changes, dry skin, and rash	Supportive skin/nail care; dermatologic evaluation and dose modification for persistent or severe toxicity
Stomatitis/mucositis	Common; predominantly grade 1–2	Oral supportive care; treatment interruption or dose modification if severe or persistent

**Table 2:** Major pivotal clinical trials and clinical evidence supportive of pemigatinib use in CCA. All abbreviations employed are defined in the text in the Abbreviations section.

Trial/Study	Evidence Status	Population	Clinical Setting	Design	Treatment	Key Efficacy Findings/Current Status	Main Eligibility
FIGHT-202 (NCT02924376) [24,89]	Completed (Phase II registration study; final analysis available)	Previously treated patients with advanced, metastatic, or unresectable CCA	Second-line or later; FGFR2 fusion/rearrangement-positive CCA	Multicenter, open-label, single-arm	Pemigatinib monotherapy	In patients with FGFR2 fusions/rearrangements: ORR 37.0%; median DOR 9.1 months; median PFS 7.0 months; median OS 17.5 months. Activity was substantially lower in patients with other FGF/FGFR alterations. Results supported regulatory approval.	Adults with unresectable or metastatic CCA after ≥1 prior systemic therapy; FGFR2 fusion/rearrangement for the principal efficacy cohort, with additional cohorts for other or no FGF/FGFR alterations.
FIGHT-302 (NCT03656536) [93,94]	Completed (Phase III randomized trial)	Treatment-naïve patients with unresectable or metastatic FGFR2-rearranged CCA	First-line advanced CCA	Global, open-label, randomized, active-controlled	Pemigatinib vs. gemcitabine plus cisplatin	Pemigatinib improved median PFS (8.3 vs. 6.8 months; HR, 0.58; 95% CI, 0.39–0.87), ORR (47% vs. 15%), and median DOR (14.2 vs. 6.3 months). Median OS was similar (24.4 vs. 25.0 months). The trial was closed early following changes in the first-line standard of care; OS interpretation was also influenced by subsequent FGFR inhibitor treatment in the chemotherapy group.	Adults with unresectable or metastatic CCA with centrally confirmed FGFR2 rearrangement/fusion and no prior systemic therapy for advanced disease.
FIGHT-101 (NCT02393248) [36,86]	Completed (Phase I/II first-in-human study)	Patients with advanced solid tumors, including CCA	Advanced/refractory solid tumors	Dose-escalation and dose-expansion	Pemigatinib monotherapy and combination cohorts	Established the recommended Phase II dose, characterized safety and pharmacokinetics, and demonstrated preliminary antitumor activity, with responses enriched in tumors harboring FGFR2 fusions/rearrangements, supporting subsequent CCA-specific development.	Patients with advanced refractory malignancies enrolled in molecularly selected and non-selected cohorts; adequate organ function required.
Global Expanded Access Program [80]	Expanded-access program	Previously treated patients with locally advanced or metastatic FGFR-altered CCA	Post-trial/pre-commercial access	Expanded-access/observational	Pemigatinib monotherapy	Provided complementary evidence on the feasibility and safety of pemigatinib outside registration trials in patients without access to commercially available therapy.	Locally advanced or metastatic CCA with FGFR alterations after prior systemic therapy and without satisfactory alternative treatment options or commercial access, according to program criteria.
Real-world pemigatinib studies [128,129,130,131,141]	Retrospective/observational evidence	Patients with FGFR2-positive advanced CCA treated in routine practice	Routine clinical practice	Real-world observational cohorts	Pemigatinib monotherapy	Reported clinical activity and a safety profile broadly consistent with FIGHT-202 while extending evidence to more heterogeneous patient populations and routine treatment settings.	Advanced CCA with FGFR2 fusion/rearrangement treated with pemigatinib according to routine clinical practice, generally after prior systemic therapy.

**Table 3:** Characteristic and clinically relevant adverse events associated with zanidatamab and principal management considerations according to refs. [45,46,201]. All abbreviations employed are defined in the text in the Abbreviations section.

Characteristic/Clinically Relevant Toxicity	Frequency/Clinical Relevance	Principal Management Considerations
Diarrhea	Very common; predominantly low grade, although severe events may occur	Prompt antidiarrheal treatment and hydration; treatment interruption or dose modification for persistent/severe toxicity
Infusion-related reactions	Common; usually grade 1–2 and generally occur during or shortly after infusion	Premedication and infusion-rate modification as appropriate; interrupt or discontinue treatment according to severity
Cardiac dysfunction/reduced LVEF	Uncommon but clinically important with HER2-directed therapy	Baseline and periodic cardiac assessment; treatment interruption and cardiology evaluation for clinically significant dysfunction

**Table 4:** Major clinical studies and selected real-world evidence of zanidatamab in CCA. All abbreviations employed are defined in the text in the Abbreviations section.

Trial/Study	Evidence Status	Population	Clinical Setting	Design	Treatment	Key Efficacy Findings/Current Status	Main Eligibility
HERIZON-BTC-01 (NCT04466891) [46,161,162]	Completed (pivotal Phase IIb; final analysis available)	Patients with HER2-positive, ERBB2-amplified unresectable locally advanced or metastatic BTC, including CCA	Previously treated advanced HER2-positive BTC	Multicenter, open-label, single-arm	Zanidatamab monotherapy	Final analysis: confirmed ORR 41.3%; median DOR 14.9 months; median PFS 5.5 months; median OS 15.5 months. Activity was greater in HER2 IHC 3+ tumors (ORR 51.6%; median OS 18.1 months) than in IHC 2+ disease. Results supported regulatory approval of zanidatamab in previously treated HER2-positive BTC.	Adults with unresectable locally advanced or metastatic HER2-positive/ERBB2-amplified BTC after prior gemcitabine-containing therapy; HER2 status determined according to protocol-defined criteria.
Phase I zanidatamab solid-tumor study [43,45]	Completed (Phase I)	Patients with advanced HER2-expressing or HER2-amplified solid tumors, including BTC	Advanced/refractory HER2-positive solid tumors	Dose-escalation and dose-expansion	Zanidatamab monotherapy	Established safety, pharmacokinetics, and the recommended dose and demonstrated preliminary antitumor activity across HER2-driven malignancies, providing the rationale for subsequent BTC-specific development.	Advanced HER2-expressing or HER2-amplified solid tumors refractory to or without appropriate standard therapy; adequate organ function according to protocol criteria.
HERIZON-BTC-302 (NCT06282575) [175,176]	Ongoing (randomized Phase III)	Patients with HER2-positive unresectable locally advanced or metastatic BTC, including CCA	First-line advanced HER2-positive BTC	Global, randomized, open-label, active-controlled	Zanidatamab + gemcitabine/cisplatin with or without immune checkpoint inhibition vs. standard first-line therapy	Evaluating whether addition of zanidatamab to first-line systemic therapy improves clinical outcomes in HER2-positive advanced BTC. This trial will help define the role of HER2-directed therapy earlier in the treatment course; efficacy results are pending.	Adults with HER2-positive unresectable locally advanced or metastatic BTC eligible for first-line systemic therapy; protocol-defined restrictions on prior systemic and HER2-directed treatment apply.
French real-world compassionate-access cohort [163]	Retrospective real-world/compassionate-access evidence	Patients with HER2-positive advanced BTC treated in routine practice	Compassionate-access setting after prior systemic therapy	Investigator-initiated, national, multicenter retrospective study	Zanidatamab	Reported clinical activity and manageable toxicity broadly consistent with prospective HERIZON-BTC-01 findings while extending evidence to a more heterogeneous routine-practice population.	Patients with metastatic HER2-positive BTC who received zanidatamab through compassionate access in France.

**Table 5:** Characteristic and clinically relevant adverse events associated with ivosidenib and principal management considerations according to [50,51,218,253]. All abbreviations employed are defined in the text in the Abbreviations section.

Characteristic/Clinically Relevant Toxicity	Frequency/Clinical Relevance	Principal Management Considerations
Gastrointestinal toxicity	Common; mainly low grade, including nausea, diarrhea, and abdominal discomfort	Symptomatic treatment and hydration; treatment modification for persistent or severe toxicity
QT interval prolongation	Uncommon but clinically important	ECG and electrolyte monitoring; correction of reversible risk factors and treatment modification for clinically significant QTc prolongation

**Table 6:** Major clinical studies and supportive real-world evidence of ivosidenib in CCA. All abbreviations employed are defined in the text in the Abbreviations section.

Trial/Study	Evidence Status	Population	Clinical Setting	Design	Treatment	Key Efficacy Findings/Current Status	Main Eligibility
ClarIDHy/AG120-C-005 (NCT02989857) [50,51,218]	Completed (pivotal Phase III registration trial)	Patients with previously treated IDH1-mutant advanced CCA	Previously treated unresectable or metastatic IDH1-mutant CCA	Multicenter, randomized, double-blind, placebo-controlled	Ivosidenib monotherapy vs. placebo; crossover to ivosidenib permitted after progression	Ivosidenib significantly improved median PFS (2.7 vs. 1.4 months; HR, 0.37; 95% CI, 0.25–0.54). ORR was 2.4%, reflecting predominantly disease-stabilizing activity. Median OS was 10.3 vs. 7.5 months (HR, 0.79; 95% CI, 0.56–1.12); 70% of placebo-treated patients crossed over to ivosidenib, and crossover-adjusted median OS for placebo was 5.1 months (HR, 0.49; 95% CI, 0.34–0.70). Results supported regulatory approval.	Adults with unresectable or metastatic CCA harboring a confirmed IDH1 mutation after prior systemic therapy; protocol-defined performance status and adequate organ function required.
Phase I IDH1-mutant CCA cohort (NCT02073994) [52,212]	Completed (Phase I dose-escalation/dose-expansion study)	Patients with advanced IDH1-mutant CCA	Advanced/refractory IDH1-mutant CCA	First-in-human dose-escalation and dose-expansion	Ivosidenib monotherapy	Established the recommended dose and demonstrated acceptable tolerability and durable disease stabilization, with relatively few objective responses, providing the rationale for subsequent randomized Phase III evaluation.	Patients with advanced IDH1-mutant solid tumors, including a dedicated CCA expansion cohort, whose disease had progressed following or who were unsuitable for standard therapy.
ProvIDHe study [229,254]	Phase IIIb/prospective multicenter study	Patients with previously treated IDH1-mutant advanced CCA	Post-standard-therapy/broader clinical-practice population	Multicenter, open-label Phase IIIb study	Ivosidenib monotherapy	Provided additional evidence on the safety and clinical effectiveness of ivosidenib in a broader previously treated IDH1-mutant CCA population, with findings generally consistent with the pivotal development program.	Previously treated patients with advanced IDH1-mutant CCA meeting protocol-defined molecular and clinical eligibility criteria.
Real-world ivosidenib cohorts [230,231,232,255,256,257]	Retrospective/observational evidence	Patients with IDH1-mutant advanced CCA, predominantly intrahepatic CCA	Routine clinical practice after prior systemic therapy	Real-world observational/multicenter cohorts	Ivosidenib monotherapy	Reported clinically relevant disease control, survival outcomes, and manageable toxicity broadly consistent with ClarIDHy while extending evidence to more heterogeneous routine-practice populations.	Advanced IDH1-mutant CCA treated with ivosidenib in routine clinical practice after prior systemic therapy and molecular confirmation of an IDH1 mutation.

**Table 7:** Contemporary management of CCA: clinically focused overview of current treatment strategies, including indications, representative regimens, biomarkers, toxicity considerations, and supporting evidence. The table below integrates contemporary guideline-based and biomarker-driven strategies for CCA, including recent updates and actionable molecular subsets. All abbreviations employed are defined in the text in the Abbreviations section.

Modality	Indication/When Used	Example Regimens/Agents	Key Evidence	Biomarkers/Toxicity
Surgery	Potentially curative option for localized, resectable disease	Resection with lymphadenectomy; liver-directed surgery in selected intrahepatic CCA	Surgical series and guideline recommendations [80,297]	Requires accurate staging; recurrence risk remains high
Adjuvant therapy	After curative-intent resection, especially in high-risk patients	Capecitabine	BILCAP trial supporting adjuvant capecitabine [298]	Hand–foot syndrome, diarrhea, fatigue; consider margin/nodal status
First-line systemic therapy	Unresectable locally advanced or metastatic CCA	Gemcitabine + cisplatin + durvalumab	TOPAZ-1 established chemoimmunotherapy as a first-line standard [14]	Monitor hematologic toxicity, biliary infection risk, immune-related adverse events
Alternative first-line chemotherapy	When immunotherapy is unsuitable or unavailable	Gemcitabine + cisplatin; gemcitabine + oxaliplatin	ABC-02 established gemcitabine–cisplatin survival benefit [13]	Platinum toxicity, renal function, neuropathy, myelosuppression
Second-line chemotherapy	Disease progression after first-line gemcitabine–platinum therapy	FOLFOX	ABC-06 showed modest but clinically relevant survival benefit [24]	Neuropathy, cytopenias, gastrointestinal toxicity
FGFR2-targeted therapy	Previously treated advanced intrahepatic CCA with FGFR2 fusion/rearrangement	Pemigatinib; futibatinib; infigratinib where available	FIGHT-202 and FOENIX-CCA2 support FGFR inhibition [26,260]	FGFR2 fusion/rearrangement; hyperphosphatemia, nail/skin changes, ocular toxicity
IDH1-targeted therapy	Previously treated advanced CCA with IDH1 mutation	Ivosidenib	ClarIDHy trial established benefit in IDH1-mutant CCA [46,51]	IDH1 mutation; QT prolongation, fatigue, nausea, differentiation syndrome rarely
HER2-directed therapy	HER2-positive advanced BTC/CCA after prior systemic therapy; increasingly explored earlier	Zanidatamab; trastuzumab-based combinations; trastuzumab deruxtecan in selected cases	HERIZON-BTC-01 and HER2-directed basket/phase II studies [44,163]	HER2 amplification/overexpression; diarrhea, infusion reactions, cardiotoxicity monitoring; ILD risk with ADCs
BRAF/MEK inhibition	BRAF V600E-mutant advanced CCA/BTC	Dabrafenib + trametinib	ROAR basket trial supports activity in BRAF V600E BTC [299]	BRAF V600E mutation; fever, rash, fatigue, cardiomyopathy, ocular events
MSI-H/dMMR immunotherapy	Advanced MSI-H/dMMR CCA/BTC, usually after prior therapy or when guideline-appropriate	Pembrolizumab; dostarlimab in selected settings	KEYNOTE studies and tumor-agnostic approvals [53]	MSI-H/dMMR; immune-related adverse events
NTRK fusion therapy	Rare NTRK fusion–positive advanced CCA/BTC	Larotrectinib; entrectinib	Tumor-agnostic NTRK fusion trials [300]	NTRK fusion; dizziness, fatigue, liver enzyme elevation
Liver-directed/locoregional therapy	Selected intrahepatic CCA with liver-dominant disease or oligoprogression	Ablation, transarterial therapies, radiotherapy, SBRT	Selected prospective and retrospective studies; guideline-supported in selected patients [3,301]	Liver reserve, biliary anatomy, infection risk, radiation-induced liver injury
Supportive and palliative care	Throughout disease course, particularly with biliary obstruction, cholangitis, cachexia, pain, or declining performance status	Biliary drainage/stenting, antibiotics, analgesia, nutritional support, symptom-directed care	Guideline-based multidisciplinary care [302,303,304]	Infection, cholestasis, quality of life, treatment tolerance

## Data Availability

No new data were created or analyzed in this study. Data sharing is not applicable to this article.

## Abbreviations

The following abbreviations are used in this manuscript:

2-HG	D-2-hydroxyglutarate
5-FU	5-fluorouracil
α-KG	alpha-ketoglutarate
ADC	antibody–drug conjugate
ADCC	antibody-dependent cell-mediated cytotoxicity
AKT	protein kinase B
ALT	alanine aminotransferase
AST	aspartate aminotransferase
ATP	adenosine triphosphate
BRAF	v-Raf murine sarcoma viral oncogene homolog B1
BTC(s)	biliary tract cancer(s)
CA 19-9	carbohydrate antigen 19-9
Ca^2+^	calcium ion
CBC	complete blood count
CCA	cholangiocarcinoma
CI	confidence interval
CPS	combined positive score
ctDNA	circulating tumor DNA
(d)MMR	deficient mismatch repair
DNA	deoxyribonucleic acid
DOR	duration of response
ECG ERBB2	electrocardiogram erb-b2 receptor tyrosine kinase 2
ERCP	endoscopic retrograde cholangiopancreatography
ERK(1/2)	extracellular signal-regulated kinase (1/2)
EUS	endoscopic ultrasound
FGF(s)	fibroblast growth factor(s)
FGFR(1–3)	fibroblast growth factor receptor (2)
FISH	fluorescence in situ hybridization
FOLFOX	folinic acid, fluorouracil, and oxaliplatin
FRS2	fibroblast growth factor receptor substrate 2
GDP	guanosine diphosphate
GemCis	gemcitabine plus cisplatin
GRB(1/2)	growth factor receptor-bound protein (1/2)
GTP	guanosine triphosphate
HER(2/3)	human epidermal growth factor receptor (2/3)
HR	hazard ratio
IDH1	isocitrate dehydrogenase 1
IDH1m	mutant isocitrate dehydrogenase 1
IHC	immunohistochemistry
ILD	interstitial lung disease
ISH	in situ hybridization
JAK	Janus kinase
KRAS	Kirsten rat sarcoma viral oncogene homolog
LFTs	liver function tests
LV	leucovorin (folinic acid)
LVEF	left ventricular ejection fraction
MAPK	mitogen-activated protein kinase
Me	methylation
MEK(1/2)	mitogen-activated protein kinase kinase 1/2
MET	mesenchymal–epithelial transition factor
mDOR	median duration of response
mo	months
mOS	median overall survival
mPFS	median progression-free survival
MRI	magnetic resonance imaging
MSI(-H)	microsatellite instability(-high)
mTORC1	mechanistic target of rapamycin complex 1
MUGA	multigated acquisition scan
NADP^+^	nicotinamide adenine dinucleotide phosphate (oxidized)
NADPH	nicotinamide adenine dinucleotide phosphate (reduced)
NGS	next-generation sequencing
NR	not reached
NTRK	neurotrophic tyrosine receptor kinase
ORR	objective response rate
OS	overall survival
PDK1	3-phosphoinositide-dependent protein kinase 1
PD-L1	programmed death ligand 1
PFS	median progression-free survival
PI3K	phosphoinositide 3-kinase
PKC	protein kinase C
PLCs	phospholipases C
PLCγ	phospholipase C gamma
QTc	corrected QT interval
RAF	rapidly accelerated fibrosarcoma kinase
RAS	rat sarcoma viral oncogene homolog
RET	rearranged during transfection proto-oncogene
RHEB	Ras homolog enriched in brain
SBRT	stereotactic body radiotherapy
SOS	son of sevenless homolog
SRC	SRC proto-oncogene non-receptor tyrosine kinase
STAT(3)	signal transducer and activator of transcription (3)
TAS-102	trifluridine/tipiracil
TEAE	treatment-emergent adverse event
TET	ten-eleven translocation
TMB	tumor mutational burden
TMB-H	high tumor mutational burden
TOPAZ-1	trial of durvalumab plus gemcitabine and cisplatin in advanced biliary tract cancer
TRK	tropomyosin receptor kinase
TSC(1/2)	tuberous sclerosis complex (1/2)
